# Attenuation Effect of *Withania somnifera* Extract on Restraint Stress-Induced Anxiety-like Behavior and Hippocampal Alterations in Mice

**DOI:** 10.3390/ijms26157317

**Published:** 2025-07-29

**Authors:** Kippuem Lee, Daehyeop Lee, Joo Yun Kim, Jae Jung Shim, Jae Woo Bae, Jae Hwan Lee

**Affiliations:** 1 R&BD Center, hy Co., Ltd., 22 Giheungdanji-ro, 24 Beon-gil, Giheung-gu, Yongin-si 17086, Republic of Korea; joy4917@hanmail.net (K.L.); flywhy7@hy.co.kr (D.L.); jjshim@hy.co.kr (J.J.S.); jaehwan@hy.co.kr (J.H.L.); 2 3HLABS Co., Ltd., 240, Kintex-ro, Ilsanseo-gu, Goyang-si 10391, Republic of Korea; jerry@3h-labs.com

**Keywords:** *Withania somnifera* extract, restraint stress, hippocampus, depression, anxiety

## Abstract

Stress is a major factor that threatens the body’s homeostasis or well-being. Excessive stress causes psychological anxiety and tension, which disrupts the balance of the autonomic nervous system that maintains the body’s balance, resulting in hormonal imbalance and brain changes. In this study, we investigated the effects of *Withania somnifera* (Ashwagandha) extract on depression, neurobehavior, and hippocampal changes in model mice exposed to stress. Using an excessive restraint stress-induced depression model, we measured the behavioral changes and the levels of *brain-derived neurotrophic factor* (*BDNF*) and antioxidant genes in five groups: control, stress, low-dose *W. somniferous* extract (20 mg/kg/day), high-dose *W. somniferous* extract (40 mg/kg/day), and L-theanine (50 mg/kg/day, positive control). Stressed mice showed poorer performance in the open field and elevated plus maze tests compared with the control group. The impaired performance was restored following *W. somniferous* extract administration. In addition, *W. somniferous* extract restored the decreased expression of BDNF in the hippocampus caused by restraint stress, improved the balance of stress hormones (i.e., cortisol, dopamine, and norepinephrine), and also regulated *BDNF*, inflammatory genes, and antioxidant genes in brain tissue. Therefore, *W. somniferous* extract can induce antidepressant and anti-stress effects by maintaining brain BDNF expression and preventing hippocampal tissue alterations caused by restraint stress.

## 1. Introduction

Stress is defined as a physiological, psychological, and behavioral response to internal and external pressures on the body [1]. The stress response is an organic and integrated response to stressors that threaten the body’s homeostasis or well-being, which increases susceptibility to various diseases [2]. Excessive stress causes psychological anxiety and tension, which disrupts the balance of the autonomic nervous system that maintains the body’s balance, resulting in hormonal imbalances and brain alterations [3]. Moreover, stress can cause physiological changes in the endocrine, autonomic, and immune systems, as well as psychological changes, such as memory and emotional dysfunctions [4]. This can lead to the development of various pathological diseases, such as brain tumors, neurodegenerative diseases, and neuropsychiatric diseases [5]. Given these negative effects of stress, there has been increased attention on research into interventions to maintain brain health. However, the complex nature of brain structure and function, the molecular mechanisms of neural circuits, and the characteristics of brain diseases (including genetic and environmental factors) have presented challenges for such research.

Restraint stress is an established representative stress model where the animal is placed within a tube to restrict movement, which causes unavoidable physical and psychological stress [6]. Recent reports have revealed that restraint stress induces anxiety-like behavior and stress-related behavioral changes, such as high-level anxiety during the elevated plus maze (EPM) test. Furthermore, it induces anxiety-like behavior via neurooxidative damage [7]. For example, Swiss albino mice subjected to restraint stress showed increased serum corticosterone levels and catalase (CAT) and superoxide dismutase (SOD) activity in the hippocampus, which reflect changes in oxidative stress [8]. Therefore, unavoidable physical and mental stress can cause antioxidant imbalances in the brain, which results in emotional, physical, and behavioral abnormalities.

The etiology of depression and anxiety remains poorly understood and cannot be explained by a single mechanism [9]. However, recent studies have shown that stress, often cited as a cause of depression and anxiety, induces hippocampal atrophy and decreases brain-derived neurotrophic factor (BDNF). BDNF is a neurotrophic factor and signaling protein that plays a role in various brain functions, such as neurogenesis, growth, cell survival, and synapse formation [10]. Excessive stress can deplete hippocampal BDNF and inhibit the neuroprotective cell signaling cascade by preventing BDNF from binding to its receptor. In addition, excessive stress causes morphological changes in the hippocampus [4], which is a key brain region that regulates memory and stress hormones. Indeed, hippocampal functions, such as learning and memory, are easily disturbed by stress [11]. For example, restraint stress temporarily reduces the number of dendritic spines and branches of pyramidal neurons in the hippocampus [11].

*Withania somnifera* (Ashwagandha) is a widely cultivated herb in the Indian subcontinent [12]. In traditional Indian medicine (Ayurveda), *W. somnifera* extract is classified as Rasayana, a plant-derived medicine known to help strengthen the body’s resistance to diseases and harmful environments and prolong life [13]. Owing to its various physiological functions, this herb has been used as a traditional folk remedy for immunity, aging, cancer, impotence, and infertility [14,15]. Moreover, *W. somnifera* extract has been shown to benefit quality of life, balance, stress, and sleep [16]. However, the exact dosage and efficacy of the *W. somnifera* extract for alleviating stress-induced anxiety and depression have not yet been verified. Therefore, in this study, we investigated the effects of *W. somnifera* extract, containing approximately 30 mg/g withaferin A, on the behavior and hippocampus of mice subjected to restraint stress. Withaferin A is a type of steroidal withanolide compound, one of the major components of *W. somnifera,* and has been reported to have anti-inflammatory and anticancer effects [17]. In this study, withaferin A was used as a marker rather than as a functional aspect of *W. somnifera*.

## 2. Results

### 2.1. Effect of W. somniferous Extract on Behavior in Restraint Stress-Exposed Mice

**Figure 1:**
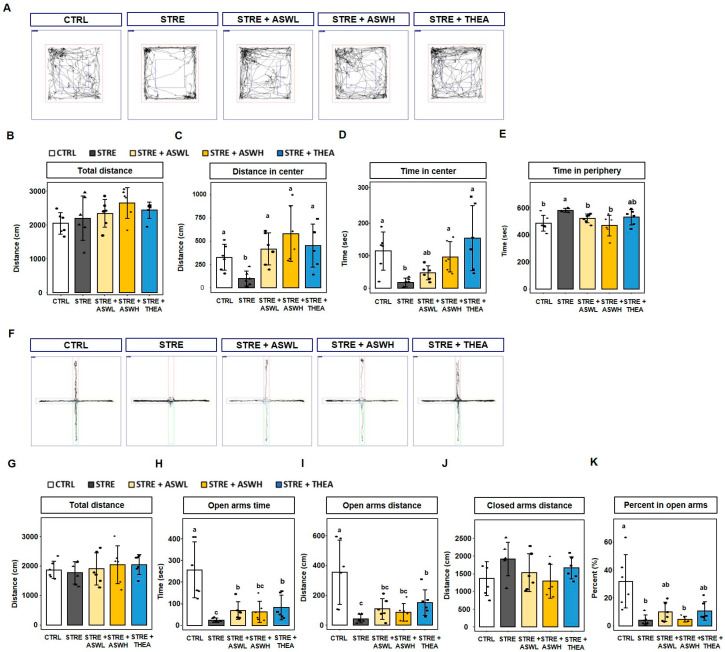
Effect of *W. somniferous* extract on anxiety and depressive behaviors in stress-exposed mice. (**A**–**E**) Representative tracks of mice in the open field test (OFT) and their frequency of entering into and time spent in the open arms (*n* = 6). Red box, entire area; blue box, center of chamber. (**F**–**K**) Representative tracks of mice in the elevated plus maze test and their frequency of entering into and time spent in the open arms (*n* = 6). The red, yellow, green and blue arms were distinguished for experiments. All data were statistically analyzed using one-way ANOVA followed by Tukey’s post hoc test. Different letters indicate significant differences (*p* < 0.05). CTRL, control mice; STRE, stress-exposed mice; STRE + ASWL, stress-exposed mice treated with 20 mg/kg/day *W. somniferous* extract; STRE + ASWH, stress-exposed mice treated with 40 mg/kg/day *W. somniferous* extract; STRE + THEA, stress-exposed mice treated with 50 mg/kg/day L-theanine.

The total distance traveled by mice in the OFT did not differ significantly among the groups (Figure 1A,B). The STRE group traveled a significantly shorter distance in the center than the CTRL group, whereas the *W. somniferous* extract and L-theanine administration groups showed a significant benefit from the treatments (Figure 1C). Specifically, the restraint stress group spent 18.4 s in the center, whereas the control group spent 114.6 s in the center (Figure 1D). However, the STRE + ASWL and STRE + ASWH groups spent significantly longer in the center compared to the STRE group (47.8 and 96.7 s, respectively). Furthermore, the STRE + THEA group spent 153.8 s in the center of the box. The STRE group spent 581.6 s in the periphery, which was significantly longer than the time spent by the CTRL group (485.4 s). Notably, the *W. somniferous* extract- and L-theanine-treated groups spent a significantly shorter time in the periphery than the CTRL group, although this difference was not significant (Figure 1E).

For the EPM test, there was no significant difference in the total distance traveled among the groups (Figure 1F,G). The time spent and distance moved in the open arms area were significantly lower in the STRE group (100 cm) than in the CTRL group (326 cm). The STRE + ASWL and STRE + THEA groups spent significantly longer in the open arms than the STRE group (68.8 cm and 82.9 cm, respectively). However, *W. somniferous* administration did not induce open arm exploration behavior to the same extent as theanine administration (Figure 1H,I). Furthermore, the distance traveled in the closed arm was lower than the STRE group in only the STRE + ASWH group (Figure 1J,K). Our data suggest that the administration of *W. somniferous* extract can improve the behavioral function of mice exposed to stressful situations.

### 2.2. Effect of W. somniferous Extract on Tissue Weight and Biochemical Parameters in Restraint Stress-Exposed Mice

**Figure 2:**
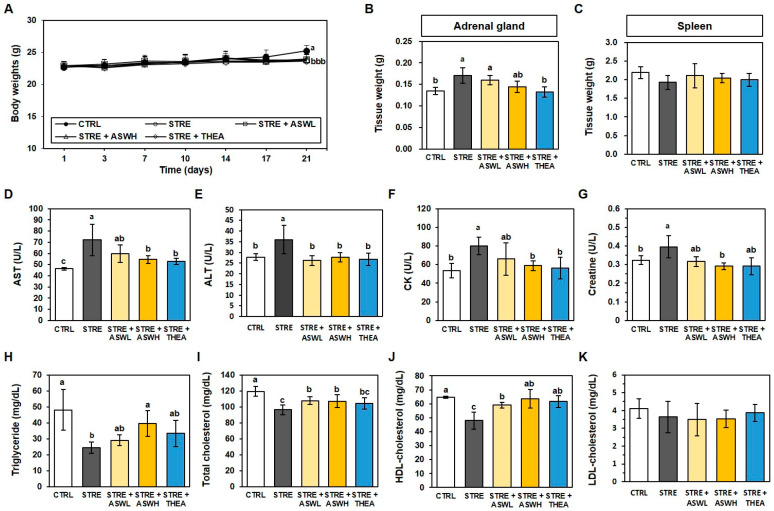
Effect of *W. somniferous* extract on body mass, tissue weight, and serum parameters. Graphs depict the measurements for each group for the following: (**A**) body mass, (**B**) adrenal gland weight, (**C**) spleen weight, (**D**) plasma aspartate aminotransferase (AST), (**E**) serum alanine aminotransferase (ALT), (**F**) creatine kinase (CK), (**G**) plasma creatinine, (**H**) triglyceride, (**I**) total cholesterol, (**J**) high-density lipoprotein (HDL) cholesterol, and (**K**) low-density lipoprotein (LDL) cholesterol. All data were statistically analyzed using one-way ANOVA followed by Tukey’s post hoc test (*n* = 6). Different letters indicate significant differences (*p* < 0.05). CTRL, control mice; STRE, stress-exposed mice; STRE + ASWL, stress-exposed mice treated with 20 mg/kg/day *W. somniferous* extract; STRE + ASWH, stress-exposed mice treated with 40 mg/kg/day *W. somniferous* extract; STRE + THEA, stress-exposed mice treated with 50 mg/kg/day L-theanine.

After the 3-week experimental period, the body weight of all restraint stress-exposed mice was significantly lower than the CTRL group (Figure 2A). In addition, the restraint stress-exposed group showed higher adrenal weights and lower spleen weights than the CTRL mice (Figure 2B). Interestingly, *W. somniferous* extract did not affect body weight or spleen weight, but the STRE + ASWH group showed a significantly lower adrenal mass than the CTRL group. As shown in Figure 2D–G, the restraint stress group exhibited significantly higher levels of AST, ALT, CK, and creatine (72.0, 36.0, 80.0, and 0.40 U/L, respectively) than the CTRL group (46.4, 27.8, 53.6, and 0.32 U/L, respectively). However, the high-dose *W. somniferous* extract and L-theanine groups showed lower levels of these biomarkers compared to the STRE group. In addition, the concentrations of triglyceride, total cholesterol, and HDL cholesterol in the STRE group (24.3, 96.3, and 47.9 mg/dL) were significantly lower than those in the CTRL group (118.0, 119.3, and 64.6 mg/dL), as shown in Figure 2H–K. Both *W. somniferous* extract groups had significantly higher levels of these lipid biomarkers than the STRE group, whereas the L-theanine group exhibited significantly higher levels of triglycerides and HDL cholesterol only. LDL cholesterol did not differ significantly among any of the groups (Figure 2K).

### 2.3. Effect of W. somniferous Extract on Serum Stress Hormone Levels in Restraint Stress-Exposed Mice

**Figure 3:**
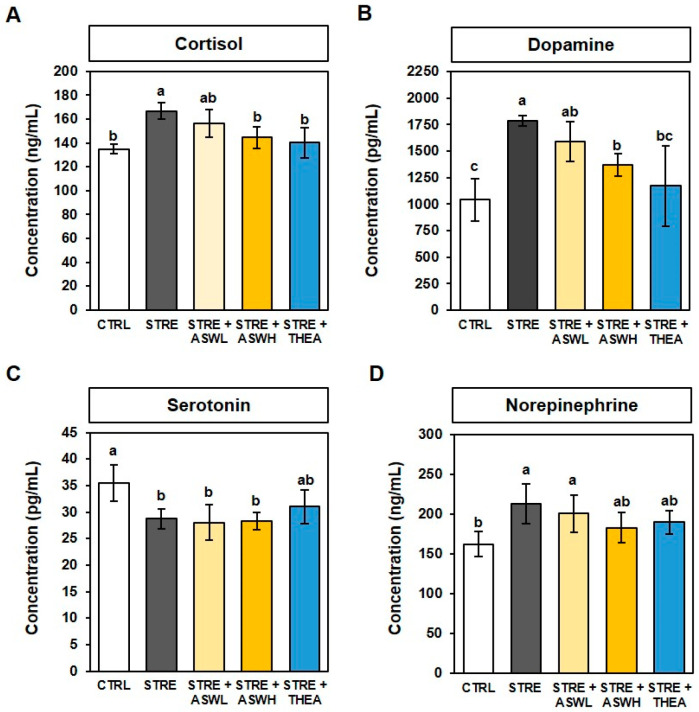
Effect of *W. somniferous* extract on blood stress hormone concentration. (**A**) Cortisol, (**B**) dopamine, (**C**) serotonin, and (**D**) norepinephrine concentrations were detected using a commercial colorimetric enzyme-linked immunosorbent assay kit. All data were statistically analyzed using one-way ANOVA followed by Tukey’s post hoc test (*n* = 6). Different letters indicate significant differences (*p* < 0.05). CTRL, control mice; STRE, stress-exposed mice; STRE + ASWL, stress-exposed mice treated with 20 mg/kg/day *W. somniferous* extract; STRE + ASWH, stress-exposed mice treated with 40 mg/kg/day *W. somniferous* extract; STRE + THEA, stress-exposed mice treated with 50 mg/kg/day L-theanine.

As shown in Figure 3A,B, the cortisol and dopamine levels were significantly higher in the STRE group (123% and 171.5%, respectively) than in the CTRL group. Levels of these two hormones were significantly lower in the high-dose *W. somniferous* extract (86.7% and 76.8%, respectively) and L-theanine (84.3% and 65.7%, respectively) groups than in the STRE group. Serotonin concentration was significantly lower in the STRE group (80.9%) than in the CTRL group. In the experimental groups (*W. somniferous* extract and L-theanine groups) there was less of an effect on the serotonin levels in CTRL mice (Figure 3C). Serum norepinephrine levels in the STRE group were 130% higher than that of the CTRL group. Only the high-dose *W. somniferous* extract treatment group showed a significantly lower level (86.0%) than the STRE group (Figure 3D). These data indicate that *W. somniferous* extract alleviates the aberrant changes in blood stress hormones caused by restraint stress.

### 2.4. Effect of W. somniferous on Hippocampal Formation and BDNF Expression in the Hippocampus of Restraint Stress-Exposed Mice

**Figure 4:**
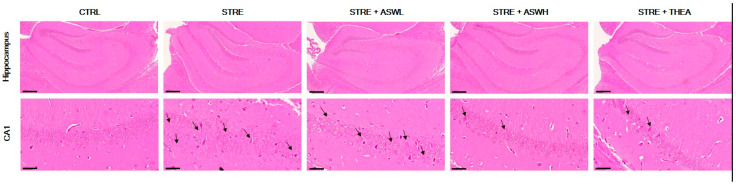
Hematoxylin and eosin staining of the total hippocampus and the cornu ammonis 1 (CA1) region of the hippocampus in mice (magnification: hippocampus 40×; CA1 200×). Black arrows indicates pathological changes such as neuronal cell loss, nuclei shrinkage, or cerebral edema. CA1, cornu ammonis 1; CTRL, control mice; STRE, stress-exposed mice; STRE + ASWL, stress-exposed mice treated with 20 mg/kg/day *W. somniferous* extract; STRE + ASWH, stress-exposed mice treated with 40 mg/kg/day *W. somniferous* extract; STRE + THEA, stress-exposed mice treated with 50 mg/kg/day L-theanine.

Figure 4 shows marked morphological changes in the hippocampus. We detected neuronal cell loss, nuclei shrinkage, and cerebral edema in the hippocampal CA1 region of the STRE group. Additionally, the degree of chromatin aggregation was higher in the STRE group compared to the CTRL mice. However, the *W. somniferous* extract treatment groups showed fewer pathological changes in a dose-dependent manner. Specifically, the aggregation and composition in the hippocampus CA1 region appeared to normalize with the administration of 40 mg/kg/day of *W. somniferous* extract to the same degree as in the L-theanine-treated mice.

**Figure 5:**
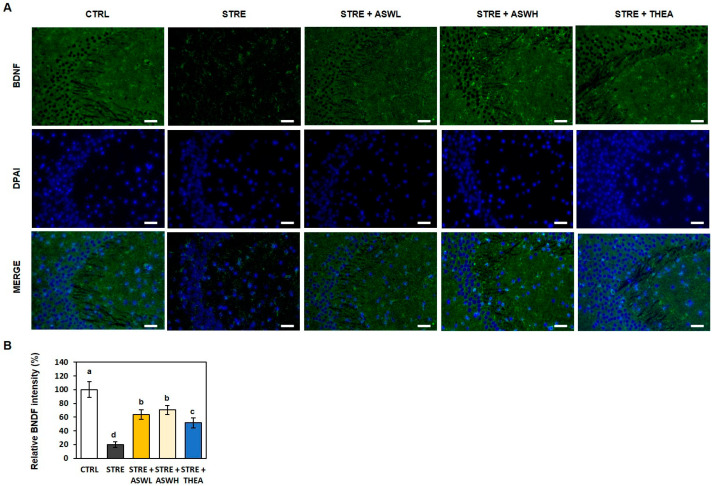
The effect of *W. somniferous* extract on brain-derived neurotrophic factor (BDNF) expression in mice. (**A**) Representative image of brain-derived neurotrophic factor (BDNF) immunofluorescence (green) and DAPI (blue) in the hippocampus of mice (magnification: 400×) and (**B**) quantitative data of BDNF intensity. CTRL, control mice; STRE, stress-exposed mice; STRE + ASWL, stress-exposed mice treated with 20 mg/kg/day *W. somniferous* extract; STRE + ASWH, stress-exposed mice treated with 40 mg/kg/day *W. somniferous* extract; STRE + THEA, stress-exposed mice treated with 50 mg/kg/day L-theanine. All data were statistically analyzed using one-way ANOVA followed by Tukey’s post hoc test (*n* = 3). Different letters indicate significant differences (*p* < 0.05).

We also confirmed the effect of *W. somniferous* extract on BDNF expression in the CA3 region of the hippocampus using IF staining (Figure 5). The STRE group showed significantly lower BDNF expression in the CA3 region than the CTRL mice, as shown by a significantly lower BDNF fluorescence intensity (green). However, the *W. somniferous* extract and L-theanine groups showed a significantly higher BDNF fluorescence intensity than the STRE mice. Therefore, *W. somniferous* extract administration may reverse stress-induced structural changes and impaired BDNF expression in the hippocampus.

### 2.5. Effect of W. somniferous Extract on mRNA Levels for BDNF, Inflammation, and Antioxidants in Restraint Stress-Exposed Mice

**Figure 6:**
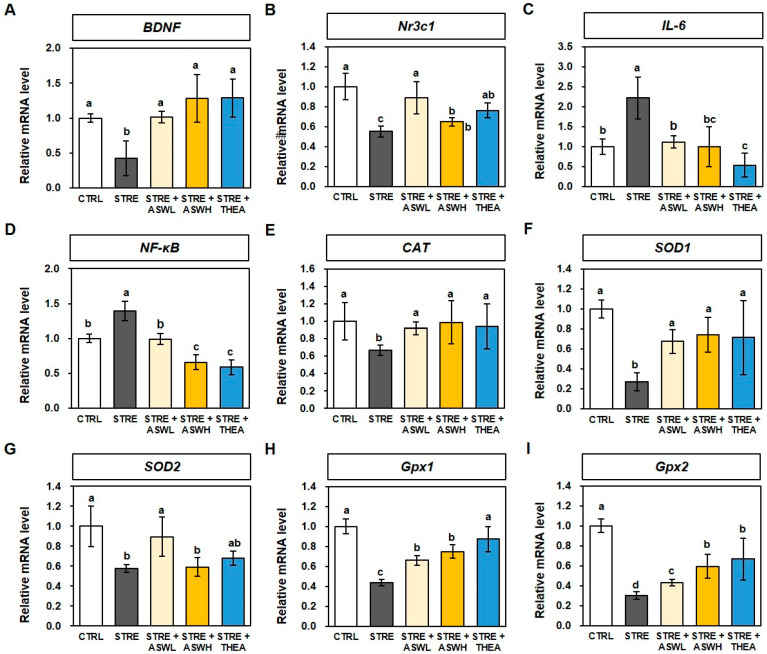
The effect of *W. somniferous* extract on brain-derived neurotrophic factor (BDNF), pro-inflammatory, and antioxidant factors in mice. The messenger RNA (mRNA) levels of (**A**) BDNF, (**B**) nuclear receptor subfamily 3C member 1 (*Nr3c1*), (**C**) interleukin-6 (*IL-6*), (**D**) nuclear factor kappa-light-chain-enhancer of activated B cells (*NF-κB*), (**E**) catalase (*CAT*), (**F**) superoxide dismutase 1 (*SOD1*), (**G**) superoxide dismutase 2 (*SOD2*), (**H**) glutathione peroxidase 1 (*Gpx1*), and (**I**) glutathione peroxidase 2 (*Gpx2*) were normalized to the level of glyceraldehyde-3-phosphate dehydrogenase mRNA and calculated as a relative-fold value. All data were statistically analyzed using one-way ANOVA followed by Tukey’s post hoc test (*n* = 6). Different letters indicate significant differences (*p* < 0.05). CTRL, control mice; STRE, stress-exposed mice; STRE + ASWL, stress-exposed mice treated with 20 mg/kg/day *W. somniferous* extract; STRE + ASWH, stress-exposed mice treated with 40 mg/kg/day *W. somniferous* extract; STRE + THEA, stress-exposed mice treated with 50 mg/kg/day L-theanine.

The mRNA levels of BDNF and corticoid receptor *Nr3c1* genes were 0.42- and 0.55-fold lower, respectively, in the STRE group than in the CTRL mice. However, BDNF mRNA levels in the STRE + ASWL and STRE + ASWH groups were 1.01- and 1.28-fold higher, respectively, and Nr3c1 mRNA levels were 0.89- and 0.65-fold higher, respectively. The L-theanine group also showed significantly higher gene levels than the STRE group (Figure 6A,B). For the levels of inflammatory cytokine genes *IL-6* and *NF-κB,* the STRE group showed 2.21- and 1.40-fold higher gene levels, respectively, than the CTRL group. The *W. somniferous* extract groups had significantly lower levels of the two inflammatory cytokines than the STRE group in a dose-dependent manner, although this effect was slightly smaller than that of L-theanine (Figure 6C,D). For the antioxidant-related genes, the *CAT* gene level, which plays an important role in protecting neurons from oxidative stress, was 0.67-fold lower in the STRE group compared to the CTRL group. However, both the low- and high-dose *W. somniferous* extract groups showed 0.92- and 0.99-fold higher levels of the gene, respectively, than the STRE group (Figure 6E). In addition, the low-dose *W. somniferous* extract group showed 0.67- and 0.90-fold higher levels of *SOD1* and *SOD2* genes, respectively, than the STRE group (which exhibited 0.27- and 0.58-fold lower levels, respectively, than the CTRL group; Figure 6F,G). Finally, the levels of *Gpx1* and *Gpx2* genes, which produce key antioxidant defense system enzymes, were 0.43- and 0.3-fold lower in the STRE group than in the CTRL group. However, both gene levels were significantly higher in the two *W. somniferous* extract groups in a dose-dependent manner (0.66- and 0.43-fold at 20 mg/kg/day and 0.75- and 0.59-fold at 40 mg/kg/day for the *Gpx1* and *Gpx2* genes, respectively). The L-theanine group also showed significantly higher *Gpx1* and *Gpx2* gene expression than the STRE group (Figure 6H,I).

## 3. Discussion

*W. somniferous* extract is known to have antioxidant, anti-inflammatory, anti-tumor, and immunomodulatory effects [18]. However, studies investigating the effect of *W. somniferous* extract on stress and depression caused by restraint stress have not yet been conducted. Therefore, we aimed to evaluate the effects of *W. somniferous* extract on restraint stress in 7-week-old C57BL/6 male mice. In addition, we used L-theanine as a positive control, which is known to help reduce anxiety and stress and improve cognitive function. L-theanine is known as a naturally occurring amino acid analog found in green tea [19]. It can modulate neurotransmitter levels and alleviate anxiety, and thus has been used in stress and anxiety experiments [20,21]. Stress was induced by applying the restraint stress protocol to mice for 2 h per day for 2 weeks, and *W. somniferous* extract was orally administered in saline at concentrations of 20 mg/kg/day and 40 mg/kg/day for a total of 21 days. In this study, we used male mice to reduce hormonal variability and improve group consistency. In the case of females, the number of animals required is increased due to the estrous cycle and subsequent hormonal fluctuations, requiring careful experimental design [22]. Therefore, additional studies to confirm the effects of HY7715 through these gender-specific limitations and future studies may be necessary.

The body weight of mice subjected to restraint stress was significantly lower than control mice. Stress is well known to affect body weight and food intake [23]. Numerous studies have reported that restraint stress suppresses weight gain and food intake in rodents [24]. Given that loss of appetite is one of the representative symptoms of depression, anxiety behavior induced by restraint stress due to confinement may have resulted in weight loss. However, in our study, neither *W. somniferous* extract nor L-theanine appeared to restore the weight loss caused by restraint stress. Nonetheless, our data showed that the serum triglyceride, total cholesterol, and HDL cholesterol levels of the restraint stress-exposed mice were significantly lower than those of the control mice. Serum triglyceride levels were dose-dependently higher in the *W. somniferous* extract groups and were similar to those of control mice, whereas the L-theanine group exhibited lower levels than the *W. somniferous* extract group. In addition, the *W. somniferous* extract group had significantly higher total cholesterol and HDL cholesterol levels, whereas LDL cholesterol was not affected by either restraint stress or *W. somniferous* extract. We also confirmed that restraint stress increased the weight of the adrenal gland, which is an organ that secretes the stress hormone cortisol in response to stress [25]. Changes in cortisol levels induce appetite loss and metabolic changes related to body weight [26]. Furthermore, stress decreased the weight of the spleen, which secretes hormones in various health situations. However, high concentrations of W. somniferous extract made a significant difference in the increase in spleen weight due to stress. In summary, *W. somniferous* extract may not restore weight loss caused by restraint stress; however, it may contribute to hormonal homeostasis by regulating blood lipid levels and adrenal size.

We then examined the effect of *W. somniferous* extract on the regulation of liver parameters in mice exposed to restraint stress. Our findings showed that the levels of plasma ALT and AST were significantly higher in the restraint stress group than in the control group. However, when *W. somniferous* extract was orally administered, plasma ALT and AST levels were similar to those of the control group. In addition, the *W. somniferous* extract groups showed a lower and dose-dependent level of CK and creatine than the stressed mice, which showed significantly higher levels than the control mice. The level of blood CK is increased by stress, which can cause muscle damage and other pathological conditions related to energy [27]. Moreover, serum creatine levels have been shown to increase with stress-induced liver damage [27].

We also evaluated anxiety-like behavior using the OFT and EPM test by measuring the time spent and distance traveled in the central and peripheral zones in the OFT and in the open and closed arms in the EPM test. There was no significant difference in the total distance traveled by the mice during the OFT among the groups. However, significant differences were found between the control and stressed mice in terms of the time spent and distance traveled within the central and peripheral areas. The *W. somniferous* extract group spent more time and traveled a greater distance in the central zone while spending less time and traveling a shorter distance in the peripheral zone than the stressed mice. In addition, the stressed mice spent less time, traveled a shorter distance, and had a lower proportion of entries in the open arm in the EPM test than the control group. However, the low-dose *W. somniferous* extract group had a longer open arm exploration time, and the high-dose *W. somniferous* extract group traveled a shorter distance in the closed arm than the stressed group. Similarly to the OFT, there was no difference in the total distance traveled between the control group and the stressed group. Although *W. somniferous* extract did not have a significant dose-dependent effect on EPM test performance, it benefited the exploration of the open space. Although these behavioral data suggest that *W. somniferous* extract improves anxiety-like behavior induced by restraint stress, further tests of specific depressive-like behaviors are needed to determine this, such as the forced swim test (FST) and tail suspension test (TST), which are used to evaluate depressive-like behaviors in animal studies. Depression and anxiety are caused by stress increasing due to cortisol hormone production [28]. Cortisol is a stress hormone that belongs to the glucocorticoid hormone family and is secreted from the adrenal cortex [29]. In addition, continuous exposure to stress negatively affects the central nervous system by reducing the sensitivity of dopamine receptors, which leads to the excessive production of dopamine [29], and in turn, anxiety and helplessness [30,31]. Stress also contributes to hippocampal neuronal loss as well as alterations in dopamine and dopamine metabolite levels [32,33]. Additionally, the corticotropin-releasing factor system has been shown to stimulate serotonin and norepinephrine pathways in the central nervous system during stressful situations, both of which are associated with symptoms of anxiety and depression [34]. We confirmed that restraint stress increases serum cortisol, dopamine, and norepinephrine, and decreases serotonin. We observed that the high-dose *W. somniferous* extract group had significantly lower concentrations of cortisol, dopamine, and norepinephrine than the stressed mice, and this effect was smaller in the low-dose group. By contrast, serum serotonin concentration was not affected by either *W. somniferous* extract or L-theanine. Recent studies have reported that weight changes are related to changes in certain hormone levels. Indeed, increased cortisol levels have been reported to cause appetite loss and weight-related metabolic changes [26]. This is consistent with reports demonstrating that the cortisol-producing adrenal glands increase in size under stressful situations, which can be regulated by *W. somniferous* extract. Our findings suggest that restraint stress acts as a neurotoxic agent on the hormonal system by accelerating stress hormone imbalances and neuronal damage, and these effects can be inhibited by *W. somniferous* extract.

Excessive stress can cause structural damage to the hippocampus, which can lead to cognitive dysfunction. The hippocampus regulates stress responses and is thus impacted by stress [35]. In this study, we examined the hippocampal tissue of stressed mice following *W. somniferous* extract administration and measured the expression levels of BDNF in the hippocampus using H&E and IF staining. The CA1 and CA3 regions of the hippocampus are known to play an important role in learning and spatial memory abilities [36]. We observed neuron loss, abnormal chromatin aggregation, and neuromorphological damage in the CA1 region of the mouse hippocampus exposed to stress. Compared to the stress group, neuronal morphology was significantly more normal in both the low- and high-dose *W. somniferous* extract groups; moreover, the shape and structure of the cells and the degree of chromatin aggregation were superior. To further explore the reason for the anxiety- and depression-like behavior in the stressed mice, we measured the levels of BDNF in the mouse brains using IF. BDNF belongs to the neurotrophic factor family and plays an important role in cell differentiation, neuronal survival, neuronal development, and synaptic plasticity [37]. Numerous studies have shown that the pathogenesis of depression involves a decrease in the level of BNDF [38]. Indeed, we observed lower BDNF fluorescence levels in the hippocampus of restraint stress-exposed mice. However, the *W. somniferous* extract treatment group showed higher BDNF fluorescence intensity than the stressed mice at levels similar to that of the L-theanine-treated mice. Therefore, *W. somniferous* extract may improve anxiety and depressive behavior by preventing hippocampal structural changes and BNDF inactivation due to excessive restraint stress.

Recent studies have reported that when stress hormone levels rise, the brain’s glucocorticoid receptors become dysregulated, which has been shown to be related to the dysregulation of cortisol [29]. Stress activates the hypothalamic–pituitary–adrenal axis, which induces the secretion of glucocorticoid hormones, such as cortisol. Cortisol then binds to the Nr3c1 receptor, which affects systemic physiological functions and behavior [39]. In this study, we found that the levels of BDNF and Nr3c1 genes in the brain decreased following exposure to restraint stress and the onset of anxiety and depression symptoms. However, the group treated with *W. somniferous* extract showed significantly higher levels of BDNF and Nr3c1, which suggests that *W. somniferous* extract can prevent stress-induced brain damage. The expression levels of anti-inflammatory and pro-inflammatory cytokines in brain tissue are also investigated. Numerous studies have demonstrated that nuclear NF-*κ*B cells control inflammatory responses by regulating the gene expression of inflammatory cytokines, such as IL-6, and are a central axis of intracellular signaling systems [40]. Restraint stress significantly increased the mRNA expression of IL-6 and NF-*κ*B genes in the brain tissue of mice. The mRNA expression of IL-6 and NFkB was restored by *W. somniferous* extract, which suggests that *W. somniferous* extract alleviates stress and inhibits brain damage. Additionally, mice that were administered *W. somniferous* extract had significantly lower gene levels of antioxidant enzymes, which included CAT, SOD1, SOD2, Gpx1, and Gpx2, than those exposed to restraint stress. Notably, a low dose of *W. somniferous* extract (20 mg/kg/day) had a greater effect than a high dose (40 mg/kg/day), which indicates that even a low dose of *W. somniferous* extract can sufficiently induce anti-stress effects by regulating the mRNA levels of BDNF, Nr3c1, anti-inflammatory cytokines, and antioxidants in brain tissue. Furthermore, our data suggest that *W. somniferous* extract prevents brain damage by regulating BDNF expression and inflammation and antioxidant effects in brain tissue induced by stress.

## 4. Materials and Methods

### 4.1. Animals and Experimental Design

**Figure 7:**
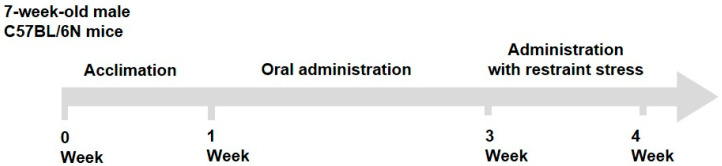
A schematic diagram of the animal experimental design.

Seven-week-old male C57BL/6N mice (DBL, Eumseong-gun, Chungcheong-do, Republic of Korea) were maintained in a controlled environment at a temperature of 19.0–25.0 °C, a relative humidity of 30–70%, and a 12 h light/dark cycle. The mice were acclimatized for 1 week with ad libitum access to water and food (Teklad Certified Irradiated Global 18% Protein Rodent Diet 2018C; Envigo RMS, Inc., Indianapolis, IN, USA). The mice were randomly divided into five groups (*n* = 8 mice/group): control (CTRL), non-restraint stress + saline; stress (STRE), restraint stress + saline; low-dose *Withania somniferous* extract (STRE + ASWL), restraint stress + *W. somniferous* extract (20 mg/kg/day); high-dose *W. somniferous* extract (STRE + ASWH), restraint stress + *W. somniferous* extract (40 mg/kg/day); and positive control (STRE + THEA), restraint stress + L-theanine (50 mg/kg/day, positive control). We used *W. somnifera* (Ashwagandha) extract containing about 30 mg/g of Withaferin A obtained from Unicorn Natural Products Pvt. Ltd. (Hyderabad, Telangana, India). Briefly, *W. somnifera* was extracted with 70% ethanol at 60 °C for 12 h, filtered, and concentrated by vacuum evaporation. For the in vivo study, the concentrate was dried in a vacuum desiccator to maintain the moisture content below 5% and the final power was used. The saline and samples were administered once daily for 21 consecutive days. From days 14 to 21 of administration, mice were subjected to restraint stress for 2 h using a restrainer (Figure 7). During this period, the saline and samples were administered 1 h before the restraint stress. Behavioral tests were conducted 30 min after the restraint stress. The open field test (OFT) was conducted on day 20, and the EPM test was performed on day 21 of administration. The mice were euthanized using carbon dioxide gas in a chamber after the behavioral test on day 21. The adrenal glands, spleen, blood, and brain were collected for further analysis. The adrenal glands and spleen were weighed, and blood samples were centrifuged at 3000 rpm for 15 min to separate the serum. The hippocampus was dissected from the brain, and all collected tissues were stored at −80 °C until analysis.

### 4.2. Behavioral Tests

#### 4.2.1. OFT

The OFT was used to evaluate locomotor activity and anxiety-like behavior evoked by stress in the mice and comprised a white acrylic apparatus (41 cm × 41 cm × 40 (h) cm). The mice were placed in the central square (20.5 cm × 20.5 cm) of the box, and behavior was recorded using the SMART 3.0 video tracking software (Panlab, Harvard Apparatus, Cornella, Spain) for 10 min. The total distance traveled, the distance traveled in the center, the time spent in the center, and the time spent in the periphery were evaluated.

#### 4.2.2. EPM Test

The EPM test comprised a white acrylic maze that consisted of two open and closed arms (arm dimensions: 76 cm × 5 cm). The closed arms had 15 cm high walls at the sides and the end, and the maze was placed at a height of 50 cm above the floor. The mice were placed in the central square (5 cm × 5 cm) facing the open arm and allowed to explore the maze for 10 min. Behavior was recorded using the SMART 3.0 video tracking software. The total distance traveled, the time spent in the open arms, the distance traveled in the open and closed arms, and the proportion of time spent in the open arms were evaluated.

### 4.3. Analysis of Biological Parameters

#### 4.3.1. Measurement of Biochemical Components in Serum

From the prepared serum, we measured aspartate aminotransferase (AST), alanine aminotransferase (ALT), creatine kinase (CK), creatine, triglyceride, total cholesterol, high-density lipoprotein (HDL) cholesterol, and low-density lipoprotein (LDL) cholesterol concentrations.

#### 4.3.2. Measurement of Cortisol, Dopamine, Serotonin, and Norepinephrine in Serum

We quantified the levels of cortisol (MyBioSource, San Diego, CA, USA; #MBS704879), dopamine (#MBS704183), serotonin (#MBS1601042), and norepinephrine (#MBS1603771), in the serum according to the manufacturer’s instructions. Absorbance was measured using a BioTek^®^ Synergy HTX multimode reader (Agilent, Santa Clara, CA, USA).

#### 4.3.3. Quantitative Reverse Transcription Polymerase Chain Reaction (qRT-PCR) Analysis

qRT-PCR was performed to evaluate gene messenger RNA (mRNA) expression in response to restraint stress. Total RNA was extracted from the brain using the Easy-spin Total RNA Extract Kit (iNtRON Biotechnology, Seoul, Republic of Korea). Complementary DNA (cDNA) was synthesized from 2 μg RNA on a thermal cycler (Bio-Rad, Hercules, CA, USA) using the Omniscript Reverse Transcription Kit (Qiagen, Hilden, Germany). The synthesized cDNA was analyzed via qRT-PCR using TaqMan^TM^ Gene Expression Assays (Applied Biosystems, Carlsbad, CA, USA). The genes used in this study were as follows: glyceraldehyde-3-phosphate dehydrogenase (*GAPDH*; Mm99999915_g1), *BDNF* (Mm04230607_s1), nuclear receptor subfamily 3C member 1 (*Nr3c1*; Mm00433832_m1), interleukin-6 (*IL-6*; (Mm01211445_m1), nuclear factor kappa-light-chain-enhancer of activated B cells (*NF-κB*; (Mm00476361_m1), catalase (*CAT*; Mm00437992_m1), superoxide dismutase 1 (*SOD1*; Mm01344233_g1), superoxide dismutase 2 (*SOD2*; Mm01313000_m1), glutathione peroxidase 1 (*Gpx1*; Mm04207457_g1), and glutathione peroxidase 2 (*Gpx2*; Mm01286848_gH). To compare mRNA levels among groups, relative mRNA levels were calculated using the 2^−ΔΔCT^ method, and relative mRNA expression data were normalized to *GAPDH*.

### 4.4. Histological and Immunofluorescence (IF) Analyses

#### 4.4.1. Hematoxylin and Eosin (H&E) Staining

The hippocampus was fixed with 10% paraformaldehyde solution at room temperature for 24 h. The tissues were embedded in paraffin, and the resulting blocks were sliced into 4 μm sections. The sectioned tissues were subsequently stained with H&E. The overall morphology and the cornu ammonis 1 (CA1) regions of the hippocampus were observed using a Nikon Eclipse E600 microscope (Nikon Corporation, Tokyo, Japan).

#### 4.4.2. IF Staining

The hippocampus sections were washed three times with Tris-buffered saline (TBS; EBA-1101, Elpisbio, Daejeon, Republic of Korea) at room temperature. Antigen retrieval was performed using Target Retrieval Solution Citrate pH 6 (S2369, DAKO, Carpinteria, CA, USA). The sections were then blocked in phosphate-buffered saline at 37 °C for 2 h using 10% goat serum (G9023, Sigma-Aldrich, St. Louis, MO, USA) and 0.3% Triton X-100 (X100, Sigma-Aldrich, USA) to block nonspecific binding sites. After blocking, sections were incubated overnight at 4 °C with a primary antibody, an anti-BDNF antibody (1:500, ab108319, Abcam, Cambridge, UK). After three washes in TBS, the sections were incubated with a fluorescent secondary antibody, Alexa Fluor 488 (1:500, ab150077, Abcam, UK), for 1 h at room temperature in the dark. Nuclei were counterstained with 4′,6-diamidino-2-phenylindole (ab104139, Abcam, UK) for 5 min, followed by a wash in distilled water. Sections were mounted using a fluorescence-compatible mounting medium and cover-slipped. Fluorescent images were acquired using a ZOE Fluorescent Cell Imager (Bio-Rad, Hercules, CA, USA).

### 4.5. Statistical Analysis

All statistical data were analyzed by using SPSS (version 20.0, IBM, Inc., Armonk, NY, USA). Data are expressed using the mean ± standard deviation (SD). Datasets were compared through one-way ANOVA, followed by Tukey’s test. *p* < 0.05 was considered to indicate statistical significance.

## 5. Conclusions

Our findings indicate that *W. somniferous* extract suppresses psychological stress in restraint stress-exposed mice by regulating stress hormones, such as cortisol, dopamine, and norepinephrine. Our results suggest that *W. somniferous* extract not only increases the expression of BDNF but also regulates the mRNA levels of inflammatory cytokines (i.e., IL-6 and NF-ĸB) and antioxidant genes (CAT, SOD1, SOD2, GPx1, and GPx2) in brain tissue. Therefore, treatment with *W. somniferous* extract may alleviate the aberrant behavioral and biochemical changes induced by restraint stress in mice, and in turn, exert antidepressant-like effects that benefit cognitive health.

## Data Availability

All data are contained within the article.

## Abbreviations

The following abbreviations are used in this manuscript:

CAT	catalase
SOD	superoxide dismutase
BDNF	brain-derived neurotrophic factor
CTRL	control mice treated with saline
STRE	restraint-stressed mice treated with saline
STRE + ASWL	restraint-stressed mice treated with *W. somniferous* extract (20 mg/kg/day)
STRE + ASWH	restraint-stressed mice treated with *W. somniferous* extract (40 mg/kg/day)
STRE + THEA	restraint-stressed mice treated with L-theanine (50 mg/kg/day, positive control)
OFT	open field test
EPM	elevated plus maze
AST	aspartate aminotransferase
ALT	alanine aminotransferase
CK	creatine kinase
HDL	high-density lipoprotein
LDL	low-density lipoprotein
GAPDH	glyceraldehyde-3-phosphate dehydrogenase
Nr3c1	nuclear receptor subfamily 3C member 1
IL-6	interleukin-6
NF-κB	nuclear factor kappa-light-chain-enhancer of activated B cells
GPx	glutathione peroxidase
H&E	hematoxylin and eosin
